# Lubricin Protects the Temporomandibular Joint Surfaces from Degeneration

**DOI:** 10.1371/journal.pone.0106497

**Published:** 2014-09-04

**Authors:** Adele Hill, Juanita Duran, Patricia Purcell

**Affiliations:** 1 Department of Orthopaedic Surgery, Boston Children's Hospital, Boston, Massachusetts, United States of America; Department of Genetics, Harvard Medical School, Boston, Massachusetts, United States of America; 2 Department of Plastic and Oral Surgery, Boston Children's Hospital and Harvard Medical School, Boston, Massachusetts, United States of America; IGBMC/ICS, France

## Abstract

The temporomandibular joint (TMJ) is a specialized synovial joint essential for the mobility and function of the mammalian jaw. The TMJ is composed of the mandibular condyle, the glenoid fossa of the temporal bone, and a fibrocartilagenous disc interposed between these bones. A fibrous capsule, lined on the luminal surface by the synovial membrane, links these bones and retains synovial fluid within the cavity. The major component of synovial fluid is lubricin, a glycoprotein encoded by the gene proteoglycan 4 (*Prg4*), which is synthesized by chondrocytes at the surface of the articular cartilage and by synovial lining cells. We previously showed that in the knee joint, *Prg4* is crucial for maintenance of cartilage surfaces and for regulating proliferation of the intimal cells in the synovium. Consequently, the objective of this study was to determine the role of lubricin in the maintenance of the TMJ. We found that mice lacking lubricin have a normal TMJ at birth, but develop degeneration resembling TMJ osteoarthritis by 2 months, increasing in severity over time. Disease progression in *Prg4* ^−/−^ mice results in synovial hyperplasia, deterioration of cartilage in the condyle, disc and fossa with an increase in chondrocyte number and their redistribution in clusters with loss of superficial zone chondrocytes. All articular surfaces of the joint had a prominent layer of protein deposition. Compared to the knee joint, the osteoarthritis-like phenotype was more severe and manifested earlier in the TMJ. Taken together, the lack of lubricin in the TMJ causes osteoarthritis-like degeneration that affects the articular cartilage as well as the integrity of multiple joint tissues. Our results provide the first molecular evidence of the role of lubricin in the TMJ and suggest that *Prg4* ^−/−^ mice might provide a valuable new animal model for the study of the early events of TMJ osteoarthritis.

## Introduction

The temporomandibular joint (TMJ) is a specialized synovial joint essential for the mobility and function of the mammalian jaw, including nutritional intake and communication. The TMJ is composed of the mandibular condyle, the glenoid fossa of the temporal bone, and an intra-articular fibrocartilagenous disc that lies between the two bones. A fibrous capsule, lined on the luminal surface by the synovial membrane, connects these two bones and encapsulates the synovial fluid within the joint cavity [1].

The epiphyses of the condyle and fossa are covered by articular cartilage, which together with the joint disc and the synovial fluid permit a smooth movement of the jaw. With age, the articular surfaces are prone to degeneration, due to intense daily use, frequently resulting in osteoarthritis (OA), a common but debilitating disorder that affects all synovial joints including the TMJ. In osteoarthritis chondrocytes respond to biomechanical and biologic stresses, resulting in breakdown of the matrix and structural changes in the underlying bone [2], [3]. OA affects the integrity of the multiple tissues that form the joint, including synovium, bone, ligaments, supporting musculature and fibrocartilaginous structures [4].

Although disorders of the TMJ, including OA, have been previously documented [5], [6], [7], no studies have focused on the role of lubricin, a major component of the synovial fluid. Lubricin studies have been aimed at its mechanical role in the TMJ [8], [9]. Synovial fluid lubricates the joint and protects the articular cartilage surfaces from erosion and protein deposition. Lubricin is a large proteoglycan encoded by the gene proteoglycan 4 (*Prg4*) and is essential in the boundary lubrication of the knee articular surfaces to maintain joint integrity [10]; however its specific role in the TMJ has not been determined.

Patients with the autosomal recessive disorder camptodactyly-arthropathy-coxa vara-pericarditis syndrome (CACP) caused by mutations in *PRG4* have joints that appear normal at birth, but over time develop severe degeneration of the cartilage surface and synoviocyte hyperplasia leading to precocious joint failure [11]. This indicates that lubricin has a dual role in synovial joints: cartilage protection and inhibition of synovial cell outgrowth [12].

In this study we report the phenotype of the TMJ in mice lacking the Prg4 gene. The TMJ in these animals presents changes comparable to those previously described for the knee joint [10], and are analogous to histopathological findings described for human TMJ-OA, the most common degenerative joint disease of the TMJ [5], [13]. The etiology of TMJ-OA is unknown, although host adaptive factors (i.e age, systemic illness, and hormones) and mechanical factors (i.e trauma, parafunction, malocclusion, overloading and increased joint friction) may all play a role [14]. Due to the difficulty in studying this disease in humans, degeneration of the TMJ has been poorly characterized. Recently several mouse models have been reported which are attributable to mutations in components of extracellular matrix; however no studies have described deletion of synovial fluid components [15], [16], [17], [18], [19], [20].

The studies described herein provide the first molecular evidence that lubricin, a major component of the synovial fluid, is essential in the maintenance of the TMJ by protecting the articular cartilage surfaces and regulating synovial cell growth. Furthermore, the *Prg4* ^−/−^ mouse may provide a new animal model for the study of early events of OA-like degeneration in the TMJ.

## Materials and Methods

### Animals

All mice were housed and all experiments were conducted in compliance with protocols approved by the Institutional Animal Care and Use Committee of Boston Children's Hospital. Mice were sacrificed by CO_2_ inhalation. Homozygous *Prg4* ^−*/*−^ and age-matched heterozygous *Prg4^+/^* ^−^ mice were generated as reported [10]. Jaws from heterozygous mice were indistinguishable from *wild-type* mice and were used as controls. For each genotype and age, heads were hemi-sected and TMJs were isolated from mice at 2 *Prg4* ^−*/*−^ (n = 16), *Prg4^+/^* ^−^ (n = 8); 4 *Prg4* ^−*/*−^ (n = 8), *Prg4^+/^* ^−^ (n = 10); 6 *Prg4* ^−*/*−^ (n = 8), *Prg4^+/^* ^−^ (n = 8); and 9 months of age *Prg4* ^−*/*−^ (n = 6), *Prg4^+/^* ^−^ (n = 6).

### Histology

Skin and brain tissue were removed and heads were fixed in 4% paraformaldehyde overnight, decalcified in 14% EDTA in PBS, pH 7.5 for 14 days and embedded in paraffin or O.C.T. compound. Sections (8–10 µm) were stained with hematoxylin/eosin, and adjacent sections were stained with Safranin O or tartrate resistant acid phosphate (TRAP), following standard procedures.

### Osteoclast quantitation

For quantitation, an image of the TMJ stained for TRAP (20X) was divided into 120 equal units; multinucleated TRAP+ cells (>2 nuclei) were considered osteoclasts.

### Immunohistochemistry

Paraffin-embedded sections of the TMJ at 2, 6, and 9 months of age were stained with rabbit polyclonal antibody against aggrecan neopeptide at 1∶200 dilution (NB-100-74350, Santa Cruz Biotechnology). Sections were reacted with biotinylated secondary antibody (Jackson Laboratories, West Grove, PA) and color was developed with ImmPact NovaRED peroxidase substrate (Vector Labs, Burlingame, CA).

### *In situ* hybridization

*In situ* hybridization was performed on frozen or paraffin sections using digoxigenin (DIG)-labeled probes and color detected with BM purple (Roche, IN, USA), as previously described [21], [22]. The Prg4 mouse probe used was generated by PCR (nucleotides 2370–3070). Images were captured on a Nikon Eclipse 80i microscope with a Spot RT3 camera.

## Results

### Loss of *Prg4* causes age-related degeneration of the TMJ

**Figure 1:**
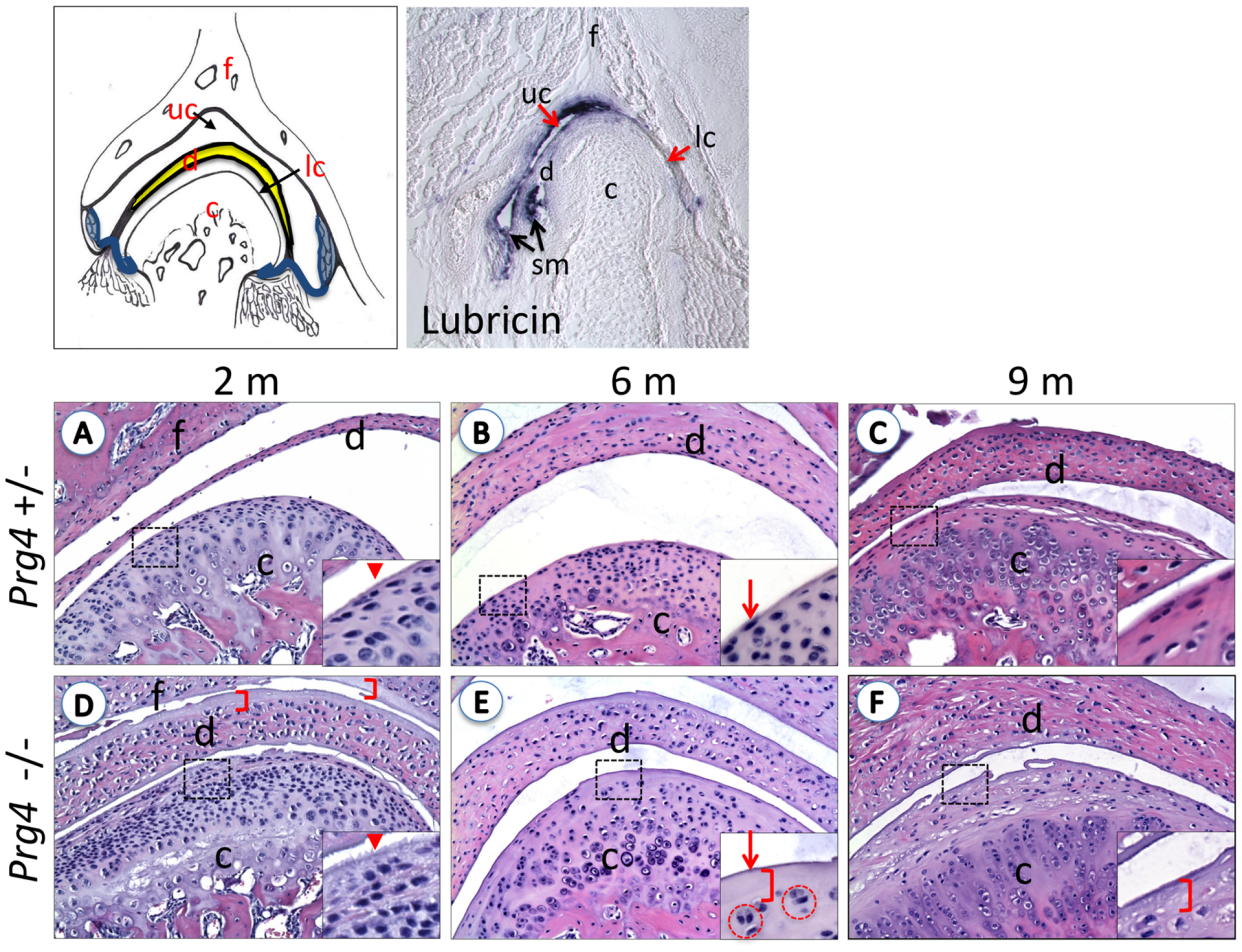
Morphological changes of the TMJ resembling osteoarthritis-like characteristics in Lubricin null (*Prg4* ^−*/*−^) mice. **Top Panels**: TMJ diagram indicating the different components of the TMJ. C: condyle, f: fossa, d: disc, uc: upper joint cavity, lc: lower joint cavity. Highlighted in blue the synovial membrane (sm). On the right, lubricin *in situ* hybridization shows localization of the *Prg4* gene primarily in the synovial membrane and upper joint cavity in mice at postnatal day 1 (10× magnification). (**A–F**) Representative coronal sections of the TMJ of lubricin control (*Prg4^+/^* ^−^) (A–C) and null (*Prg4* ^−*/*−^) (D–F) mice at 2-, 6- and 9-months of age, stained with H&E. *Prg4* ^−*/*−^ articular surfaces are irregular (arrowheads), there is an increased number of chondrocytes (dark purple dots) in the condyle (c), increased chondrocyte clusters (red circles in E), decreased number of flat cells in the most superficial layer (arrows) of the condyle, disc (d) and fossa (f), and deposit of cartilage and cellular detritus over the different structures of the joint (brackets). Thickness and cellularity in the disc progressively increases in the *Prg4* ^−*/*−^ mice compared to *Prg4^+/^* ^−^ control mice. Pictures are taken with 20× magnification and insets are 40X.

To determine the role of lubricin in the development and maintenance of the mouse TMJ, we examined sections of the TMJ of *Prg4* ^−*/*−^ and age-matched control *Prg4^+/^* ^−^ mice at embryonic day 16, birth, and 2, 4, 6 and 9 months postnatally. Embryonic and newborn *Prg4* ^−*/*−^ mice had joints of normal appearance, comparable to those of control mice (data not shown). However, by 2 months of age the joints of *Prg4* ^−*/*−^ mice displayed signs of joint degeneration when compared to control (*Prg4^+/^* ^−^) mice (Fig. 1, A vs D). In controls, the surfaces of the mandibular condyle and the glenoid fossa were smooth with flattened chondrocytes distributed across the articular surface (Fig. 1A, inset). The disc exhibited no signs of degeneration and the characteristic biconcave shape was preserved (Fig. 1A). In contrast in *Prg4* ^−*/*−^ mice the articular surfaces were irregular, the superficial flattened chondrocytes were located at a distance from the surface and fewer flattened cells were observed (Fig. 1D, inset). Additionally, a weakly stained material that appeared to represent protein deposition was observed on all articular surfaces including the condyle, the fossa and both sides of the disc (Fig. 1D brackets).

Four-month-old *Prg4* ^−*/*−^ mice had a very similar phenotype, and thus we did not study this time point further. In mutant mice of 6 and 9 months of age, fewer superficial chondrocytes were seen at the cartilage surface, and clusters of chondrocytes could be distinguished, characteristic of joint degeneration in osteoarthritis (Fig 1. E and F insets). As early as 6 months, *wild-type* mice presented early signs of naturally occurring TMJ-OA with areas of acellularity and some chondrocyte clustering [23]. However, flat superficial chondrocytes were still observed across the surface of the condyle and in general the joint appeared normal with smooth surfaces and no evidence of protein deposition (Fig. 1B).

**Figure 2:**
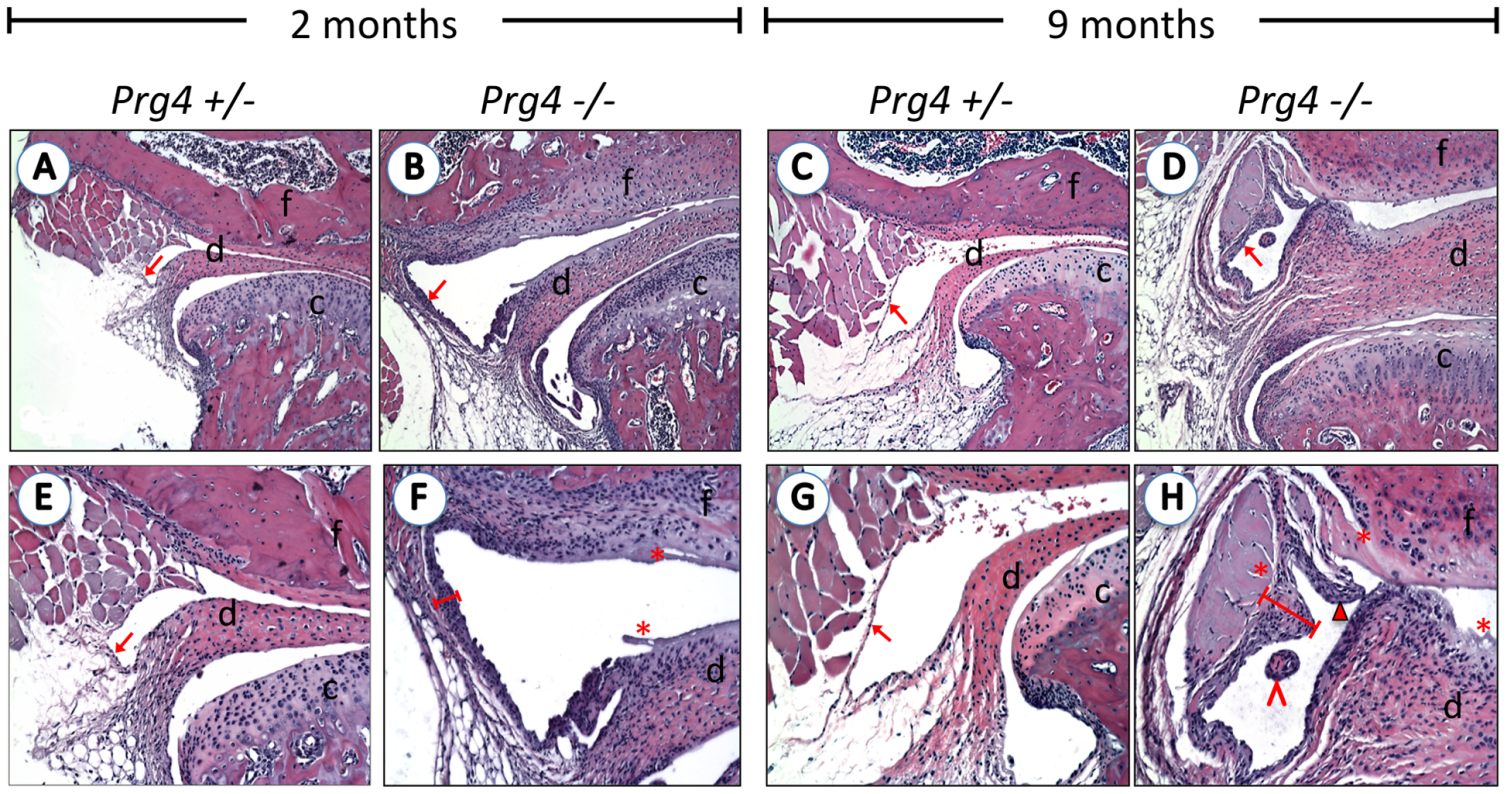
Synovial hypertrophy in *Prg4* ^−^^*/*−^ mice TMJs. TMJ sections stained for H&E at 2- and 9-months old mice in control (*Prg4* ***^+/^*** ^−^) and lubricin null mice *(Prg4* ^−***/***−^). Synovial membranes in the upper joint cavity are indicated by arrows. Control *Prg4* ***^+/^*** ^−^ mice exhibit a thin synovial lining (A, C, E, and G) compared to the hypertrophied synovium (red bar) observed in *Prg4* ^−***/***−^ mice (B, D, F, and H) and characteristic of synovitis. An increase in the severity of synovitis is observed over time, as shown in 2-month-old *Prg4* ^−***/***−^ (B, F) versus 9-month-old *Prg4* ^−***/***−^ (F, H) mice. Villous digitations (arrowhead), cartilage debris surrounded by synovial membrane (open arrowhead) and detritus rich zones (*) are observed in 9-month-old *Prg4* ^−***/***−^ mice. These inflammatory changes were seen primarily in the upper joint cavity, located between the disc (d) and fossa (f). (A–D: 10X, E–H: 20X).

At 9 months, *Prg4* ^−*/*−^ mice joints showed evidence of disease progression. Chondrocytes in the superficial layer of the condyle were scarce and the disc had a markedly increased thickness compared to controls. Cartilage tears were observed at the surfaces (Fig.1 C vs F). In addition, there was marked hyperplasia of the synovium (Fig. 2C, 2D). The articular surface of the condyle was almost completely absent, and the area of synoviocyte infiltration extended to the layer of columnar chondrocytes, especially in the center of the cartilage surface, likely due to synovial infiltration from both sides of the joint (Fig.1F). Tears in the cartilage were observed at the surface and there was almost complete loss of superficial zone chondrocytes.

### Prg4 regulates synovium growth

We studied RNA expression of *Prg4* by *in situ* hybridization at embryonic day 18 (E18), postnatal day 1 (P1) and adult TMJ (2, 6 and 9 months). We found that at all three stages of the growing TMJ, *Prg4* was strongly expressed in the synovial lining cells of the upper joint cavity and in the synovium of both edges of the TMJ, suggesting a major role for lubricin at these sites (Fig. 1 top panel) [24]. In control *Prg4^+/^* ^−^ mice, a thin single cell synovial lining, similar to that found in *wild-type* mice, was observed (Fig. 2 A, C, E and G). In contrast, a thickened synovium was seen in *Prg4* ^−*/*−^ mice (Fig. 2 B, D, F and H), characteristic of synovitis. Over time, a remarkable increase in the severity of synovial hypertrophy was apparent, as observed by comparing 2-month-old to 9-month-old *Prg4* ^−*/*−^ mice (Fig. 2 F and H). In addition, 9-month mutants presented with villous digitations (Fig. 2 H arrowhead), cartilage debris surrounded by synovial membrane (open arrowhead) and detritus rich zones (*). These inflammatory changes were seen primarily in the upper joint cavity, between the disc and the fossa where lubricin was normally expressed most abundantly (Fig. 1).

### TMJs of *Prg4* ^−*/*−^ mice show osteoarthritis-like changes

**Figure 3:**
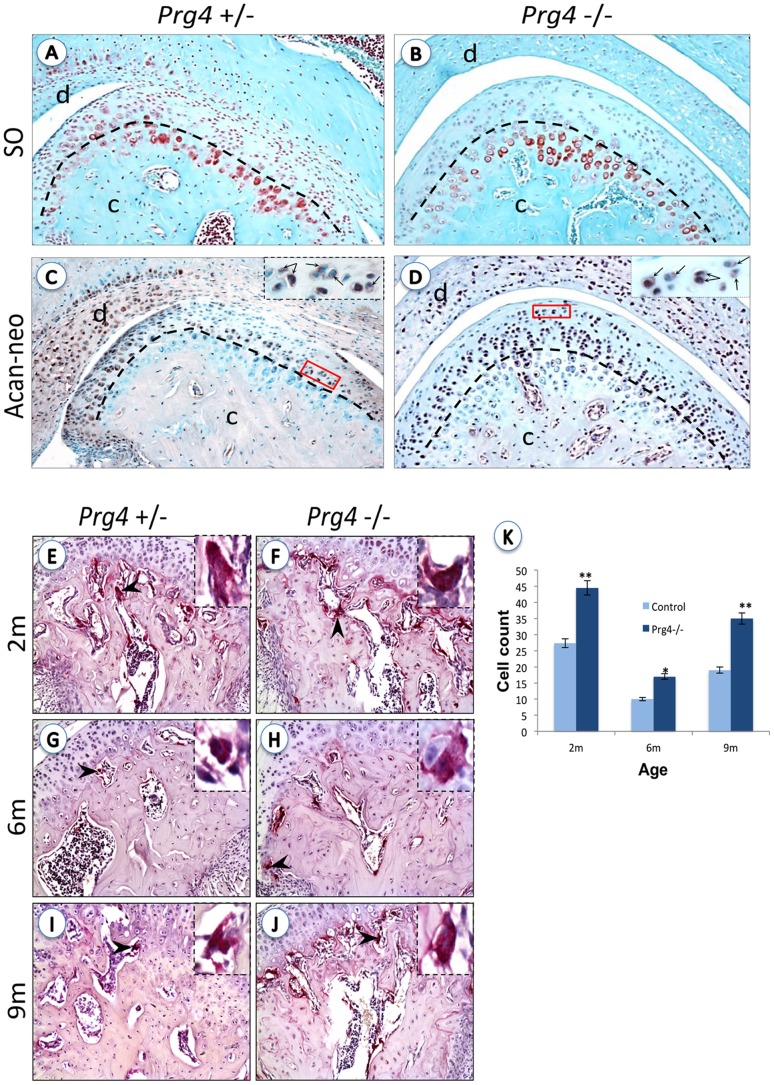
Increased TMJ Osteoarthritis-like characteristics in *Prg4* ^−^^*/*−^ mice. (**A–D**) Decreased extracellular matrix components in *Prg4* ^−***/***−^ mice. Paraffin embedded, formalin fixed TMJ sections at 6-months, histologically stained for Safranin O (SO) (A, B) and immunostained for aggrecan neopeptide (C, D) in *Prg4* ***^+/^*** ^−^ and *Prg4* ^−**/**−^ mice. A black dotted line separates the region of columnar chondrocytes in the condyle (below) from the proliferating chondrocytes (above). Note the abundant SO positive staining (red dots) in the proliferating zone in control *Prg4* ***^+/-^*** mice and the few positive SO staining in *Prg4* ^−***/***−^ null mice. As expected, the opposite is observed for aggrecan neopeptide immuno localization, which shows abundant aggregan neopeptide in proliferating chondrocytes, indicating that in *Prg4* ^−***/***−^ mice aggrecan degradation is increased. Insets in C and D correspond to a magnification of the field indicated in the red dotted line and highlight the extracellular localization of aggrecan neopeptide staining (arrows). (**E–J**) TRAP staining in 2-, 6- and 9- month TMJ sections. Black arrowheads show TRAP+ multinucleated cells (MNC) in resorptive areas of the condyles stained in red (magnified panel on right). (**K**) Quantitation of TRAP+ MNC at 2-, 6-, and 9- month-old mice showed an increase in osteoclastogenesis of 38.65%, 41.2%, and 45.8% respectively in *Prg4* ^−***/***−^ *mice*, compared to age-matched control mice. Student t-test: *, p<0.05; **, p<0.01. Dotted lines demarcate the surfaces of the condyle and fossa. c: condyle, d: disc, f: fossa.

The glycosaminoglycan content and bone resorptive activity are two parameters used to evaluate joint osteoarthritis. For proteoglycans we analyzed sections from 6-month-old *Prg4* ^−*/*−^ mice stained with Safranin O (SO), which binds to negatively charged glycosaminoglycans. *Prg4* ^−*/*−^ mice showed reduced pericellular SO staining compared to control mice (Fig. 3, A and B). In addition, we evaluated the expression of aggrecan neopeptide, which reflects the degraded extracellular matrix proteins aggrecan and collagen [25], and found a dramatic increase in products of degradation in mutant mice (Fig 3, C and D), consistent with a reduction in the proteoglycan content of the cartilage. It is relevant to note that chondrocytes that stained positive for SO were negative for aggrecan neopeptide and vice-versa (Fig. 3 A–D).

Another characteristic of osteoarthritis is bone resorption as a result of increased osteoclast activity [2]; therefore we evaluated changes in bone resorption in *Prg4* ^−*/*−^ mice with respect to their aged-matched controls. We used the TRAP assay to measure osteoclast activity. At all time points studied, TRAP positive multi-nucleated cells were significantly increased in the subchondral bone region of the mutant *Prg4* ^−*/*−^ mice as compared to their respective age-matched control *Prg4^+/^* ^−^ mice, and these differences increased with age, (Fig. 3 E–J). TRAP activity in the mutants was elevated 39%, 41% and 46% in 2-, 6- and 9-month old mice, respectively, relative to age-matched controls (Fig. 3K). Interestingly, TRAP staining peaked at 2 months in both mutant mice and age-matched controls (Fig. 3 F, J and K), indicating that this peak activity was unrelated to the *Prg4* mutation and that greater bone resorption occurred at this earlier time point. Thus, we observed an increased osteoclast activity in the absence of the *Prg4* gene that augments with age. Taken together these results strongly suggest that lubricin protects TMJ synovial joints from degeneration, and in the absence of lubricin, mice present a premature osteoarthritis-like phenotype in the TMJ.

### TMJ degeneration is more severe than knee joint degeneration in *Prg4* ^−*/*−^ mice

**Figure 4:**
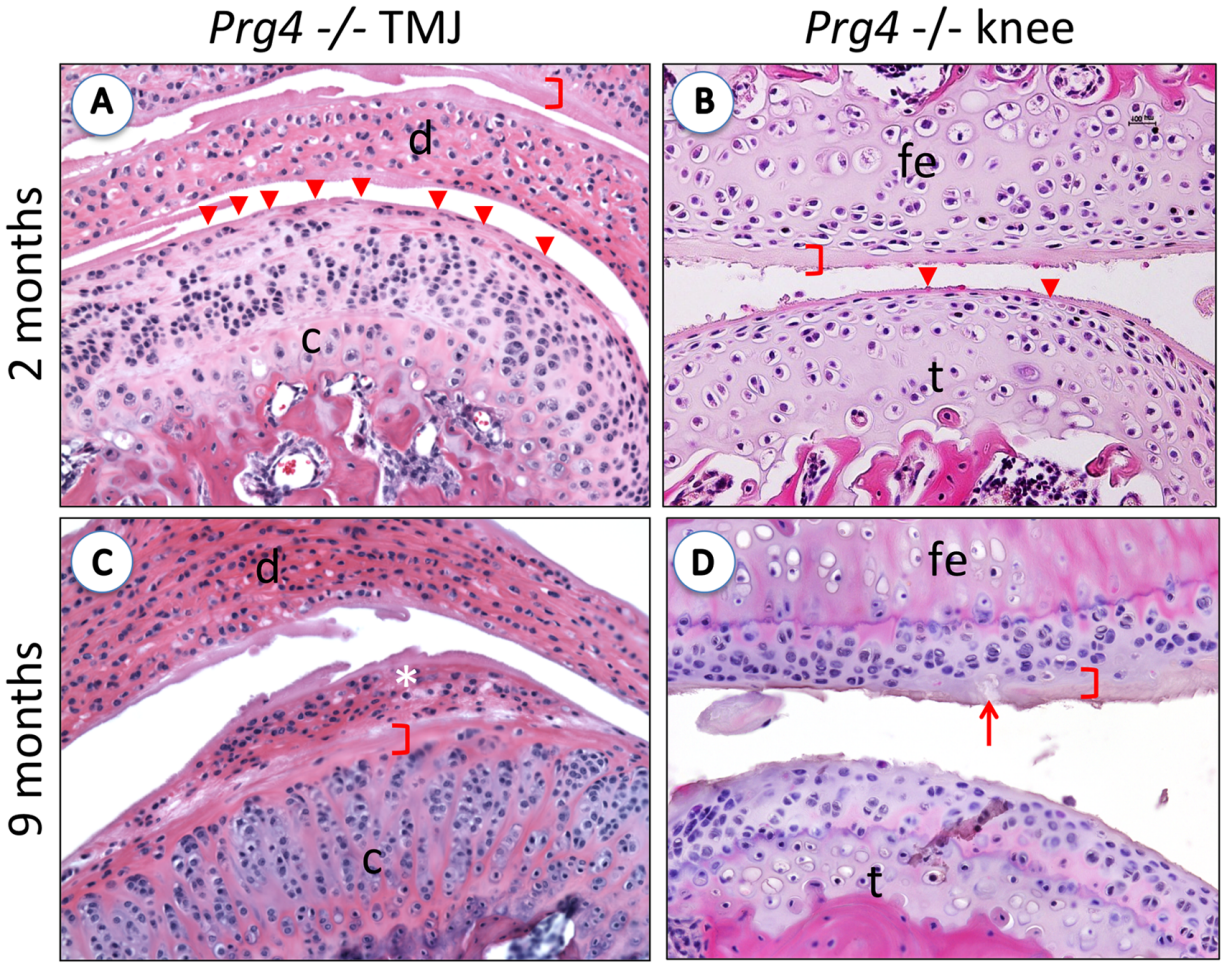
Osteoarthritis-like characteristics in TMJ and knee joints in *Prg4* ^−^^*/*−^ mice. (**A–D**) Representative coronal sections of the TMJ and knee joints of lubricin null (*Prg4* ^−***/***−^) mice at 2- and 9- months of age, stained with H&E. At 2 months, the articular surface of the TMJ condyle displays very few superficial flat chondrocytes, whereas, several superficial zone chondrocytes can still be observed at the cartilage surface in the knee joint. (A and B, arrowheads). In both joints, evidence of lightly stained protein deposition across the entire joint surfaces can be observed (Fig. 4 brackets). At 9 months of age, the cartilage surfaces of both joints display disease progression, chondrocytes are absent from the articular surfaces of both the knee and the TMJ, and the protein layer deposited on all surfaces is enlarged and disrupted (Figs. 4 C and D). In addition, in the TMJ there is a large infiltration of synoviocytes deposited on the surface of the condyle (Fig. 4C *). c: condyle, d: disc, fe: femur, t: tibia.

We previously reported that knee joints from lubricin-deficient mice appear normal at birth, but progressively degenerate over time [10]. We therefore examined the evolution of pathology in the TMJ of *Prg4* ^−*/*−^ mice compared to the knee joints of 2 and 9 month-old mice. By 2 months of age, the articular surfaces of knee joints in *Prg4* ^−*/*−^ mice showed loss of superficial zone chondrocytes, particularly from the tibial plateau where areas of acellularity were observed (Fig. 4B arrowheads). There was also evidence of protein deposition across the entire surface of both the femoral condyle and the tibial plateau (Fig. 4B brackets). Interestingly, TMJs of 2 month-old *Prg4* ^−*/*−^ mice had a much greater loss of superficial zone chondrocytes, especially on the surface of the glenoid fossa (Fig. 4A brackets). As in the articular surfaces of the knee, protein had accumulated on the surface of both the fossa and the mandibular condyle. By 9 months of age, the cartilage surfaces of both joints had worsened, superficial zone chondrocytes were not observed at the articular surface of either the knee or the TMJ, and protein deposition on the cartilage surfaces had increased (Figs. 4 C and D). In the TMJ, the entire upper layer of articular cartilage was lost and was replaced by an infiltration of synoviocytes (Fig. 4C *). In the knee, some cartilage was still observed, though there was evidence of cartilage clefts (Fig. 4D arrow), suggesting that the osteoarthritis-like phenotype observed in the TMJ was more severe than that observed for the knee joint in age-matched mice.

## Discussion

The *Prg4* ^−*/*−^ mouse is an established animal model for CACP syndrome, in which the absence of lubricin causes degenerative changes in the knee joint, resembling the fundamental features of osteoarthritis [10]. Synovial joints are the most common joint type in mammals and are characterized by the presence of synovial fluid. The TMJ is a unique synovial joint both in terms of its structure, as well as in the developmental genetic pathways that govern its formation [22]. The knee articular surfaces are covered with hyaline cartilage, whereas the TMJ articular surfaces are covered with fibrocartilage. Although TMJ-OA can be associated with arthropathies affecting other joints, few patients presenting with TMJ-OA have generalized osteoarthritis [5].

We therefore examined *Prg4* ^−*/*−^ mice to determine whether the TMJ also presents an OA-like phenotype that would, if comparable to that seen in the knee, be indicative of early changes and would allow the investigation of the first stages of TMJ-OA. We found that in adult *Prg4* ^−*/*−^ mice, the TMJ displayed significant signs of joint degeneration that increased in severity over time. Embryonic and newborn mice showed no apparent abnormalities suggesting that lubricin is not required for normal development of the TMJ, but is essential for its maintenance.

Early osteoarthritis is characterized by fibrillation and erosion of the articular cartilage surface, associated with loss of cells in the superficial zone, and protein deposition from the synovial fluid. We observed these characteristics in the TMJ of *Prg4* ^−*/*−^ mice, suggesting that a lack of lubricin induces joint degeneration that mimics human TMJ OA. Osteoarthritis is characterized by early loss of proteoglycans, leading to reduction in compressive strength of the cartilage, and subsequent joint failure [25], [26]. *Prg4* ^−*/*−^ mice exhibited loss of proteoglycan staining at all ages analyzed, and an increase in osteoclast activity (Fig. 3), hallmarks of both OA and active joint remodeling.

In addition, we observed a thickening of the disc in the TMJs of *Prg4* ^−*/*−^ mice with a loss of the characteristic bi-concave shape (Fig.1), a phenotype that has also previously been shown in human TMJ disorders [27]. This thickening may represent a defense mechanism to maintain the smoothness of the joint by compensating for the degeneration of the cartilage surface. The integrity of the disc is vital in maintaining the homeostasis of the joint, degenerative changes in the disc, including perforation, lead to disruption of the joint [28]. Disc displacements are also associated with TMJ OA [29]. Thus, our results suggest that there is inter-dependency between the integrity of the cartilage and the integrity of the disc, and that changes in either structure will have an effect on the health of the joint.

Lubricin has been shown to prevent adhesion and regulate synoviocyte cell proliferation in the knee [10]. We observed both synovial overgrowth and severe synoviocyte infiltration in the TMJ of *Prg4* ^−*/*−^ mice, most strikingly at 9 months of age (Fig. 2D, 4C), suggesting that synovial growth is also controlled by lubricin in the TMJ. The increased thickness of the disc may also be due in part to synovial infiltration. The cellular organization of the growth plate of the TMJ is not linear as it is in the knee [22], allowing for mutli- rather than uni-directional growth [23]. In addition, the articular cartilage components are different between the two joints, predominated by hyaline cartilage in the knee and fibrocartilage in the TMJ. This suggests that lubricin is able to prevent adhesion and excessive proliferation of the synovium in many joints on multiple surfaces. This information could be of particular relevance for the lubrication of engineered joint replacements.

In the mouse, the knee develops earlier than the TMJ, with joint cavitation seen at E14.5, whereas the TMJ does not develop until E16. Nevertheless there was a more severe OA-like phenotype in the TMJ than in the knee at both 2 months and 9 months in *Prg4* ^−*/*−^ mice (Fig.4), indicating that the TMJ has an earlier onset of OA-like pathology in *Prg4* ^−*/*−^ mice than the knee joint. Other TMJ-OA mouse models have been reported, for example mice double deficient in biglycan and fibromodulin, which exhibit OA of both the knees and TMJ similar to that observed in *Prg4* ^−*/*−^ mice [23], [30]. However, in these mice OA is observed in the knee at 1 month of age, whereas degeneration of the TMJ was not seen until 6 months.

In summary our results present the first structural and molecular evidence of the essential role of lubricin, the main component of the synovial fluid, in the maintenance of the integrity of the TMJ. In addition, these data also suggest that TMJ degeneration in *Prg4* ^−*/*−^ mice occurs much earlier than in other reported models [23], [30]. The *Prg4* ^−*/*−^ mouse therefore offers a distinct and valuable model for investigating an earlier onset of TMJ-OA and precocious joint failure, which in addition to the existing models will lead to a better understanding of TMJ disorders.
